# Comparison of Leisure Time Physical Activities by Metabolic Syndrome Status among Adolescents

**DOI:** 10.3390/ijerph19031415

**Published:** 2022-01-27

**Authors:** Robert Booker, Harish Chander, Keith C. Norris, Roland J. Thorpe, Brad Vickers, Megan E. Holmes

**Affiliations:** 1 Department of Preventive Medicine, Feinberg School of Medicine, Northwestern University, Chicago, IL 60611, USA; 2 Department of Kinesiology, Mississippi State University, Starkville, MS 39762, USA; hchander@colled.msstate.edu (H.C.); BVickers@colled.msstate.edu (B.V.); mholmes@colled.msstate.edu (M.E.H.); 3 Program for Research on Men’s Health, John Hopkins Bloomberg School of Public Health, Baltimore, MD 21205, USA; knorris@ucla.edu (K.C.N.); rthorpe@jhu.edu (R.J.T.J.); 4 Division of General Internal Medicine and Health Services Research, Department of Medicine, David Geffen School of Medicine, University of California, Los Angeles, CA 90095, USA; 5 Hopkins Center for Health Disparities Solutions, Johns Hopkins Bloomberg School of Public Health, Baltimore, MD 21205, USA

**Keywords:** sedentarism, exercise, metabolic syndrome, cardiometabolic risk

## Abstract

*Background:* Metabolic syndrome (MetS) increases the risk of premature morbidity and mortality. Physical activity (PA) beneficially affects MetS; however, it is unclear if PA types differ among adolescents 12–15 years old, according to their MetS status. This study compared self-reported PA types by MetS status. *Methods:* Using the 2015–2016 National Health and Nutritional Examination Survey (NHANES) data, 664 adolescents self-reported PA in the past seven days. MetS status was assessed using Ford’s pediatric adaptation of the ATP-III adult criteria. Pearson chi-square and *t*-tests were conducted to determine self-reported PA differences. *Results:* The adolescents’ mean age was 13.47 years (95% CIs; 13.04, 14.38) and 52.69% were male (352). Twenty-seven (4.07%) adolescents were MetS positive. The prevalence of PA engagement in the past seven days was similar for MetS-positive and -negative adolescents (77.67% and 70.51%, respectively; *p* > 0.05). No significant differences were observed for PA type by MetS status. MetS-positive adolescents reported higher sedentary time (565.77 [438.99, 692.56] vs. 490.59 [377.86, 603.33] minutes per day, respectively; *p* = 0239). *Conclusions:* Engagement in specific PA types does not appear to differ by MetS status, but MetS-positive adolescents have significantly higher sedentary time. PA promotion should target a variety of activities to maximize the effectiveness of public health programs and interventions should target reducing sedentary time.

## 1. Introduction

Among adolescents, the prevalence of metabolic syndrome (MetS) has been estimated to range between 0% and 19.2% [1]. Poor health during adolescence is associated with increased morbidity in adulthood [2]. MetS is a leading cause of premature morbidity and mortality and a major risk factor for cardiovascular disease [3,4,5]. Engagement in habitual physical activity (PA) is imperative to a healthy lifestyle and reduces the risk of MetS, as well as premature morbidity [6,7] and mortality [8,9,10,11,12,13,14]. The World Health Organization PA recommendations for adolescents include an average of 60 minutes daily of moderate-to-vigorous physical activity (MVPA) [15]. PA is known to decrease with increasing age [16] and weight status [17,18]. For example, participation in PA varies by type, with walking the most commonly reported PA ranging from 28–47% of adults in the United States [19,20]. Running was the second most prevalent type of PA, reported by 8–13% of United States adults [19,20]. Understanding the types of PA adolescents engage in may help to elucidate how to increase or maintain PA during the life course and the impact of such PA on cardiometabolic health.

Individuals have volition on engagement in PA and the specific type of PA. Understanding which activities adolescents engage in is useful for program and intervention efforts to increase physical activity and reduce sedentary behavior. It is currently unknown if the types of PA participated in by MetS-positive adolescents differ from MetS-negative adolescents. The purpose of the present study was to compare self-reported PA, the types of PA, and duration of PA and sedentary behavior between adolescents with and without MetS. We hypothesized the estimates for MetS-negative adolescents to have a higher prevalence overall and in each PA type compared with MetS-positive adolescents. Additionally, we hypothesized MetS-positive adolescents to have a greater amount of sedentary time compared with MetS-negative adolescents.

## 2. Methods

### 2.1. Participants

Data were gathered from the 2015–2016 NHANES cycle, a nationally representative cross-sectional survey of the non-institutionalized civilian population in the United States [21]. NHANES implements a complex, multistage probability design to recruit participants in the following steps: selection of counties or grouping of contiguous counties, selection of a small cluster of households within the previous section, selection of specific households, selection of participants within the households [22]. For the 2015–2016 NHANES cycle, a total of 15,327 participants were selected from 30 different locations. Of those 15,327 participants, 9971 completed the interview portion and 9544 completed the examination portion of data collection. NHANES routinely oversamples from specific populations of interest for health outcomes to improve the precision and reliability of the subsequent analyses. Adolescents aged 12–15 years old were included in the analysis (*N* = 664) as laboratory measures for determining MetS status were collected on individuals at least 12 years old and PA types were reported by individuals up to 15 years old.

### 2.2. Demographic Data

Demographic information, including age, sex, race, and ethnicity, was self-reported. The reported age ranged from 12–15 years old. Sex was reported as female or male. Race and ethnicity were reported as ‘Mexican American’, ‘Other Hispanic’, ‘Non-Hispanic White’, ‘Non-Hispanic Black’, ‘Non-Hispanic Asian’, and ‘Other Race—Including Multi-Racial’. ‘Mexican American’ and ‘Other Hispanic’ were collapsed into a singular group for the determination of MetS severity.

### 2.3. Laboratory Data

Blood samples were collected in mobile examination centers under controlled and standardized conditions. Participants aged at least 12 years old arrived in the morning after a nine-hour fast. The amount of blood drawn varied by age and all blood samples were stored properly before being shipped to remote laboratories for analysis [23].

### 2.4. Examination Data

Waist circumference was measured during the physical examination following standard protocols. Blood pressure was measured in triplicate after a five-minute quiet rest while sitting. The average for both systolic and diastolic blood pressure was used for the present study [24].

### 2.5. Metabolic Syndrome

Participants were categorized as MetS positive using Ford’s pediatric adaptation of the ATP-III adult criteria [25]. An individual must have any combination of at least three of the five risk factors to qualify as having MetS. The threshold for each criterion is as follows: waist circumference, ≥90th sex-specific percentile; triglycerides, ≥110 mg dL^−1^; high-density lipoprotein cholesterol, ≤40 mg dL^−1^; systolic or diastolic blood pressure ≥90th age-, height-, and sex-specific percentile; fasting blood glucose, ≥100 mg dL^−1^. MetS component data were available for adolescents aged 12 years and older. Metabolic syndrome severity *z*-scores (MetS-Z) were calculated for all participants. Sex-, race-, and ethnicity-specific formulas were used to calculate MetS-Z following previously established methodology [26]. MetS-Z uses BMI z-score, fasting blood glucose, systolic blood pressure, triglycerides, and high-density lipoprotein cholesterol to establish sex-, race-, and ethnicity-specific relative risk of MetS as *z*-scores. Only equations for Non-Hispanic White, Non-Hispanic Black, and Hispanic males and females are available. BMI *z*-scores were calculated via R (4.1.0, The R Foundation for Statistical Computing, Vienna, Austria) using the package “zscorer”.

### 2.6. Movement Behaviors

Participants self-reported the typical minutes per day spent engaged in PA by intensity based on the following question: “How much time do you spend doing vigorous-(moderate) intensity sports, fitness or recreational activities on a typical day?” Typical daily minutes of sedentary behavior was self-reported from the following question: “The following question is about sitting at school, at home, getting to and from places, or with friends including time spent sitting at a desk, traveling in a car or bus, reading, playing cards, watching television, or using a computer. Do not include time spent sleeping. How much time do you usually spend sitting on a typical day?”

### 2.7. Physical Activity Types

Participants self-reported PAs based on the following question: “For the next questions, think about the sports, lessons, or physical activities you may have done during the past 7 days? Please do not include things you did during the school day like PE or gym class. Did you do any physical activities during the past 7 days?”. Participants then reported from the following list: aerobics, baseball, basketball, bike riding, cheerleading, dance, field hockey, football, golf, gymnastics, hiking, ice hockey, ice skating, jumping rope, lacrosse, martial arts, playing games, rollerblading, running, scooter riding, skateboarding, soccer, swimming, tennis, track and field, volleyball, walking, wrestling, frisbee, backyard games, trampoline, horseback riding, and other. Participants could select multiple activities, and non-response to any of the PA types was treated as non-engagement in that specific PA type. The number of days participants played an active video game (AVG) in the past seven days was also reported based on the following question: “During the past 7 days, on how many days did you play active video games such as Wii Sports, Wii Fit, Xbox 360, Xbox Kinect, Playstation 3, or Dance, Dance Revolution?”. Responses between one day and seven days were collapsed into a dichotomous variable (Yes/No) for engagement and treated as another type of PA.

### 2.8. Statistical Analysis

Descriptive statistics were determined for the population and by MetS status. The proportion of engagement in PA type was by MetS status among those who reported engaging in PA in the past seven days. The mean and 95% confidence intervals (95% CIs) were calculated for continuous and proportion and 95% CIs for discrete variables. A series of independent sample *t-*Tests and Pearson chi-square tests were conducted to determine if the reported engagement was different between MetS status. Appropriate survey design variables were used with the survey design designated as a one-stage design survey. The most conservative sampling weight, fasting subsample 2-year MEC sampling weight, was used per NHANES procedures. One primary sampling unit was a singleton, so it was treated as a certainty unit to be able to generate 95% CIs. All analyses were conducted with significance set a priori at a threshold of *p* < 0.05 using StataSE (17.0, Stata Corp., College Station, TX, USA).

## 3. Results

Participant characteristics can be found in Table 1. Twenty-seven of the six hundred and sixty-four adolescents (4.07%) were classified as MetS-positive. MetS-positive and negative adolescents were of similar age with MetS-positive adolescents having a greater height (mean [95% CIs]; 168.01 [159.74, 176.29] vs. 161.16 [158.56, 163.75] cm, respectively; *p* = 0.0309), weight (97.92 [84.95, 110.90] vs. 58.49 [54.79, 62.20] kg, respectively; *p* < 0.0001), and BMI (34.30 [32.22, 36.39] vs. 22.38 [21.36, 23.41] kg·m^−2^, respectively; *p* < 0.0001). Regarding the individual MetS component variables, MetS-positive adolescents had significantly worse values compared with MetS-negative adolescents. Among all MetS-positive adolescents, none were positive for all five components. MetS-Z was significantly greater among MetS-positive adolescents compared with MetS-negative adolescents (1.43 [1.10, 1.76] vs. −0.21 [−0.44, 0.01], respectively; *p* < 0.0001). Both MetS-positive and -negative adolescents self-reported similar minutes per day of moderate and vigorous PA but were significantly different regarding daily sedentary time (490.59 [377.86, 603.33] vs. 565.77 [438.99, 692.56] minutes per day, respectively; *p* = 0.0239).

### Type of Physical Activity

The prevalence of engagement in each type of PA is reported in Table 2. MetS-positive and -negative adolescents self-reported a similar engagement in PA in the past seven (77.67% [44.67, 93.75] and 70.51% [61.83, 77.91]; *p* = 0.3019). MetS-positive and -negative adolescents reported engagement in ten and 26 types of PA respectively. Football (50.30% [18.91, 81.46]) was the most common MetS-positive adolescents while basketball (27.30% [17.87, 39.33]) was the most common among MetS-negative adolescents. There were no differences in prevalence in engagement across any type of PA.

## 4. Discussion

Contrary to our hypothesis, MetS-positive and -negative adolescents reported similar prevalence for engaging in PA in the past seven days. Supporting our other hypotheses, MetS-positive adolescents engaged in more sedentary time, although PA duration, both moderate and vigorous intensity, did not differ. Overall, the past seven-day PA engagement did not differ by MetS status, but MetS-positive adolescents reported engaging in 16 fewer types of PA than MetS-negative adolescents. We were surprised that the overall variety of engaged types of PA would vary by MetS status, provided total PA duration was the same for both groups.

Most of the prior research examining the type of PA engagement has focused on the adult population [19,20,27]. Due to the paucity of literature regarding the type of PA engagement among adolescents, adult PA engagement may begin to provide possible rationale. As PA engagement is known to decrease as individuals age [15], it is important to examine what future types of PA adolescents may engage in as adults. Spees and colleagues [17] observed overweight and obese adults report engaging in fewer types of PA than normal-weight adults. A lower prevalence among overweight and obese individuals was observed for aerobics, bike riding, and running than normal-weight individuals. However, reported walking prevalence did increase with weight status [17]. The high prevalence of football participation among MetS-positive adolescents relative to other activities is particularly noteworthy. Previous research has observed excess body fat [28] and high BMI [29] in adolescent football linemen. Given the high attrition of participation in this sport throughout life and into adulthood, champions of physical literacy [30] may find young football players an important target of interventions. It has been suggested that obese participants engage in low-impact types of PA or incorporate PA as a part of daily activity as opposed to structured exercise [31]. In the present study, many of the activities only chosen by MetS-negative adolescents have a greater impact (e.g., dance, gymnastics, volleyball) and the nature of these PAs may be a deterrent for engagement by MetS-positive adolescents.

Team sport participation was among the most reported types of PA among all adolescents in the present study. A record 7.9 million high school students participated in school sports in 2015–2016, with the most popular sports being team sports [32]. Adolescent team sport participation, rather than individual activities, has been observed to be associated with better social and psychological health [33]. A negative association between behavioral intentions and enjoyment with the risk of dropout of team sports has been noted [34]. The influences on which type of PA an adolescent participates in vary between each individual. Researchers have identified friendship to be an important factor [35]. The PA of an adolescent’s friend was observed to be a positive predictor of that adolescent’s PA [35]. Sex differences regarding reasons to engage in PA have also been observed. Male adolescents reported having more fun than female adolescents when participating in PA where exertion reaches levels of being out of breath [36]. Stankov and colleagues found through a systematic review that overweight adolescents experience greater difficulty in generating friendships and higher levels of victimization compared with normal-weight peers being an additional barrier to PA [37]. In the present study, the high prevalence of team sports among MetS-positive adolescents may be due to high levels of friendship experienced by the current participants. However, this is outside the scope of the present study and should be investigated further.

A strength of the present study is that it is, to our knowledge, the first study investigating the engagement of specific PA types between MetS-positive and -negative adolescents. An added strength is the use of a nationally representative survey of the United States, allowing for broad generalizability. The present findings are not without limitations. Data collected as part of NHANES are cross-sectional; therefore, causality towards MetS status cannot be established. Additionally, the reasons as to why the participants selected the types of PA to participate in cannot be determined. Individuals could have a preference or physical limitation which precludes participation in specific types of PA. The NHANES PA data are self-reported and, due to recall bias, low response rate or understanding of the question may be underestimated or overestimated by the participant; however, these are commonly acknowledged limitations of survey-based PA surveillance at the national level [38]. Finally, there were few adolescents in the MetS positive group limiting inferences.

## 5. Conclusions

In the present study, we examined if self-reported PA types differed between MetS-positive and -negative adolescents. MetS status did not influence engagement in overall PA or within the type of PA. However, MetS-negative adolescents engaged in a more varied range of types of PA and had significantly lower sedentary time. Practitioners should strive to increase engagement in types of PA to gravitate adolescents who otherwise may not engage in that type of PA, thus reducing sedentary time for MetS adolescents. Creating a welcoming team-based environment could increase participation among all adolescents.

## Figures and Tables

**Table 1:** Descriptive characteristics—by metabolic syndrome status.

	Metabolic Syndrome Positive *n* = 27	Metabolic Syndrome Negative *n* = 637	*p*-Value
Age (years)	13.71 (13.04, 14.38)	13.49 (13.21, 13.76)	0.2100
Height (cm)	168.01 (159.74, 176.29)	161.16 (158.56, 163.75)	0.0309
Weight (kg)	97.92 (84.95, 110.90)	58.49 (54.79, 62.20)	<0.0001
BMI (kg m^−2^)	34.30 (32.22, 36.39)	22.38 (21.36, 23.41)	<0.0001
Waist Circumference (cm)	111.52 (100.73, 122.32)	77.24 (74.27, 80.21)	<0.0001
Triglycerides (mg·dL^−1^)	145.29 (101.62, 188.96	62.91 (57.55, 68.27)	0.0002
Fasting Blood Glucose (mg·dL^−1^)	102.25 (100.46, 104.04)	100.38 (91.4, 109.36)	0.7794
Systolic Blood Pressure (mm Hg)	117.46 (109.34, 125.57	106.50 (104.36, 108.63)	0.0067
Diastolic Blood Pressure (mm Hg)	58.66 (54.53, 62.80)	56.97 (54.75, 59.20)	0.8740
High-Density Lipoprotein (mg·dL^−1^)	41.94 (31.85, 52.04)	55.79 (53.39, 58.19)	0.0017
Number of MetS Criteria			
0		48.73% (40.81, 56.72)	
1		34.60% (26.75, 43.39)	
2		16.67% (11.36, 23.79)	
3	74.69% (47.55, 90.57)		
4	25.31% (9.43, 52.45)		
5	0%		
MetS-Z	1.43 (1.10, 1.76)	−0.21 (−0.44, 0.01)	<0.0001
Sedentary Behavior (min·day^−1^)	490.59 (377.86, 603.33)	565.77 (438.99, 692.56)	0.0239
Moderate PA (min·day^−1^)	42.76 (25.59, 59.92)	53.38 (37.57, 69.19)	0.0638
Vigorous PA (min·day^−1^)	92.03 (46.68, 137.37)	8.20 (55.92, 90.86)	0.3482

Data are presented as mean (95% CIs) or proportion (95% CIs) where appropriate. Systolic and diastolic blood pressures are the average readings per individual. BMI = Body Mass Index, MetS = Metabolic Syndrome, MetS-Z = Metabolic Syndrome Continuous Severity *z*-Score, PA = Physical Activity. Sample sizes reported are unweighted.

**Table 2:** The proportion of the type of physical activity engagement.

	Metabolic Syndrome Positive *n* = 27	Metabolic Syndrome Negative *n* = 637
Any physical activity in last seven days	77.67% (44.67, 93.75)	70.51% (61.83, 77.91)
Active Video Gaming	48.43% (17.93, 80.13)	25.79% (17.31, 36.58)
Aerobics	5.26% (0.88, 25.85)	11.53% (5.82, 21.55)
Backyard Games	-	1.08% (0.26, 4.37)
Baseball	2.39% (0.27, 18.11)	6.71% (3.05, 14.16)
Basketball	19.97% (4.77, 55.42)	27.30% (17.87, 39.33)
Bike Riding	2.87% (0.24, 26.70)	9.45% (4.28, 19.56)
Cheerleading	-	4.15% (0.54, 25.61)
Dance	-	3.10% (0.96, 9.53)
Field Hockey	-	-
Football	50.30% (18.91, 81.46)	19.83% (13.88, 27.53)
Frisbee	-	0.94% (0.22, 3.97)
Golf	-	-
Gymnastics	-	0.62% (0.06, 6.19)
Hiking	-	-
Horseback Riding	-	-
Ice Hockey	-	-
Ice Skating	-	-
Jumping Rope	-	1.17% (0.20, 6.49)
Lacrosse	-	2.62% (0.55, 11.53)
Martial Arts	-	1.45% (0.32, 6.37)
Playing Games	-	0.48% (0.05, 4.12)
Rollerblading	11.03% (1.15, 56.89)	-
Running	11.15% (2.53, 37.81)	22.71% (17.34, 29.15)
Scooter Riding	-	0.57% (0.06, 5.16)
Skateboarding	-	1.84% (0.67, 4.93)
Soccer	9.26% (2.67, 27.57)	13.26% (7.25, 23.01)
Swimming	-	3.46% (1.35, 8.59)
Tennis	-	0.45% (0.06, 3.49)
Track and Field	-	1.47% (0.38, 5.56)
Trampoline	-	4.30% (1.84, 9.72)
Volleyball	-	4.53% (1.31, 14.5)
Walking	25.09% (4.09, 72.44)	14.85% (8.02, 25.85)
Wrestling	-	0.45% (0.06, 3.51)

Sample sizes reported are unweighted. Proportion (95% CIs).

## Data Availability

All of the data used in the study are publicly available from the Centers for Disease Control and Prevention.
